# Digital Dietary Behaviors in Individuals With Depression: Real-World Behavioral Observation

**DOI:** 10.2196/47428

**Published:** 2024-04-22

**Authors:** Yue Zhu, Ran Zhang, Shuluo Yin, Yihui Sun, Fay Womer, Rongxun Liu, Sheng Zeng, Xizhe Zhang, Fei Wang

**Affiliations:** 1 Early Intervention Unit Department of Psychiatry The Affiliated Brain Hospital of Nanjing Medical University Nanjing China; 2 Functional Brain Imaging Institute of Nanjing Medical University Nanjing China; 3 School of Biomedical Engineering and Informatics Nanjing Medical University Nanjing China; 4 School of Computer Science and Engineering Northeastern University Shenyang China; 5 Department of Psychiatry and Behavioral Sciences Vanderbilt University Medical Center Nashville, TN United States; 6 Henan Key Laboratory of Immunology and Targeted Drug, Henan Collaborative Innovation Center of Molecular Diagnosis and Laboratory Medicine School of Laboratory Medicine Xinxiang Medical University Xinxiang China

**Keywords:** dietary behaviors, digital marker, depression, mental health, appetite disturbance, behavioral monitoring, eating pattern, electronic record, digital health, behavioral, surveillance

## Abstract

**Background:**

Depression is often accompanied by changes in behavior, including dietary behaviors. The relationship between dietary behaviors and depression has been widely studied, yet previous research has relied on self-reported data which is subject to recall bias. Electronic device–based behavioral monitoring offers the potential for objective, real-time data collection of a large amount of continuous, long-term behavior data in naturalistic settings.

**Objective:**

The study aims to characterize digital dietary behaviors in depression, and to determine whether these behaviors could be used to detect depression.

**Methods:**

A total of 3310 students (2222 healthy controls [HCs], 916 with mild depression, and 172 with moderate-severe depression) were recruited for the study of their dietary behaviors via electronic records over a 1-month period, and depression severity was assessed in the middle of the month. The differences in dietary behaviors across the HCs, mild depression, and moderate-severe depression were determined by ANCOVA (analyses of covariance) with age, gender, BMI, and educational level as covariates. Multivariate logistic regression analyses were used to examine the association between dietary behaviors and depression severity. Support vector machine analysis was used to determine whether changes in dietary behaviors could detect mild and moderate-severe depression.

**Results:**

The study found that individuals with moderate-severe depression had more irregular eating patterns, more fluctuated feeding times, spent more money on dinner, less diverse food choices, as well as eating breakfast less frequently, and preferred to eat only lunch and dinner, compared with HCs. Moderate-severe depression was found to be negatively associated with the daily 3 regular meals pattern (breakfast-lunch-dinner pattern; OR 0.467, 95% CI 0.239-0.912), and mild depression was positively associated with daily lunch and dinner pattern (OR 1.460, 95% CI 1.016-2.100). These changes in digital dietary behaviors were able to detect mild and moderate-severe depression (accuracy=0.53, precision=0.60), with better accuracy for detecting moderate-severe depression (accuracy=0.67, precision=0.64).

**Conclusions:**

This is the first study to develop a profile of changes in digital dietary behaviors in individuals with depression using real-world behavioral monitoring. The results suggest that digital markers may be a promising approach for detecting depression.

## Introduction

The mental health of students has become the forefront of concerns, particularly since the onset of the COVID-19 pandemic. Approximately 45% of college students in China reported experiencing mental health issues during the outbreak [1]. Depression screening typically involves self-reported data, but there is a lack of objective markers to promptly identify individuals experiencing depression. Early identification and intervention are crucial for mitigating the impact of depression during critical periods for the academic and occupational functioning of students [2].

Appetite disturbance or changes in dietary behaviors are common symptoms of depression and may serve as objective indicators of the condition in a large population [3]. Dietary behavior can exert an influence on mental health through a variety of pathways, including circadian rhythms, oxidative stress, and the gut microbiota [4]. Based on the time of meals, intervals between meals, daily eating window, and food intake of the day, dietary behavior patterns can be categorized into morningness, intermediate, and eveningness chronotypes [5]. Eveningness chronotype commonly exhibits a higher tendency to skip breakfast, eat dinner later, and allocate a greater proportion of their daily food intake to later hours of the day [6]. Prior research has additionally demonstrated that the eveningness chronotype, coupled with social jetlag, constitutes a risk factor for depression [7,8]. Besides, individuals with more pronounced fluctuations in their eating windows often display heightened emotional vulnerability [9]. However, previous studies of diet relied on retrospective questionnaires or interviews, which may fail to accurately reflect real-world behavior [10]. Additionally, these methods typically assess only 1 aspect of dietary behavior, such as diet quality or eating habits [8,11,12]. To fully understand dietary behaviors, it is necessary to use multiple scales to assess multiple dimensions, such as diet quality, emotional eating, and chronotype of eating habits. However, using multiple scales can lead to participants taking too long to complete the questionnaire, reducing its validity. Furthermore, understanding daily behavior features requires repeated behavioral monitoring over an extended period, while retrospective reports from a single point in time may not accurately reflect true behavior [13].

The use of electronic platforms for behavioral monitoring allows for real-time assessment of human behavior and can trigger an alert if measured behavior deviates from healthy norms [14,15]. Additionally, these platforms enable the collection of large amounts of high-frequency, high-dimensional continuous data, which can be used to identify typical multidimensional behavior features over an extended period based on naturalistic situations [16,17]. The growing body of literature leveraging behavioral monitoring for depression prediction has gained traction, spurred by the profound shifts in lifestyle behavior patterns, especially during the COVID-19 pandemic [18-20]. Nonetheless, predominant inquiries have predominantly concentrated on probing the correlation between physical activity, social network engagement, and mental well-being facilitated through mobile devices [21-23]. However, an evident void persists in comprehensively exploring the nexus between the surveillance of dietary behavior patterns and depression. On university campuses, meals are often paid for using electronic transactions linked to a student account, providing the opportunity to collect digital dietary behavior data. In this study, real-world monitoring was used to track dietary behavior for a month, and data on time, expenditure, and location patterns were collected.

To the best of our knowledge, this is the first study to use electronic device–based behavioral monitoring instead of retrospective self-reported data to examine the digital dietary behaviors of individuals with depression compared with controls, and to investigate the relationship between these behaviors and depression. It is common for depression to occur alongside other symptoms, but the relationship between digital dietary behaviors and these comorbid symptoms of depression remains underexplored. Therefore, we also aim to determine whether these comorbid symptoms are associated with dietary behaviors and to clarify the role these symptoms play in the relationship between changes in dietary behavior and depression. The final data analysis will involve using digital dietary behavior features to detect depression.

## Methods

### Recruitment of Participants

A total of 3678 medical students from Xinxiang Medical University willingly engaged in this study, responding to the institution’s mental health survey notification. As part of this engagement, they underwent a cross-sectional mental status survey from October 6 to 12, 2020. Concurrently, during the survey, these participants also consented to furnish records of their eating behaviors for the period spanning from October 1 to 31, 2020. These records were sourced from electronic transactions linked to their respective student accounts. All participants completed questionnaires via WeChat (Tencent Corp) and signed web-based informed consent.

### Web-Based Measurements

Basic sociodemographic characteristics, such as gender, age, BMI, and educational level, were collected using the WeChat official account platform. All participants also completed the following psychological assessments: the Patient Health Questionnaire-9 (PHQ-9), the Generalized Anxiety Disorder Questionnaire-7 (GAD-7), the Perceived Stress Scale-14 (PSS-14), and the Insomnia Severity Index (ISI). All the details of these psychological assessments can be found in Multimedia Appendix 1.

### Study Participants and Inclusion Criteria

Based on the outcomes of the psychological survey, the inclusion criteria for the mild depression group, the moderate-severe depression group, and the healthy control (HC) group, as well as the exclusion criteria, were defined as follows.

**Figure 1:**
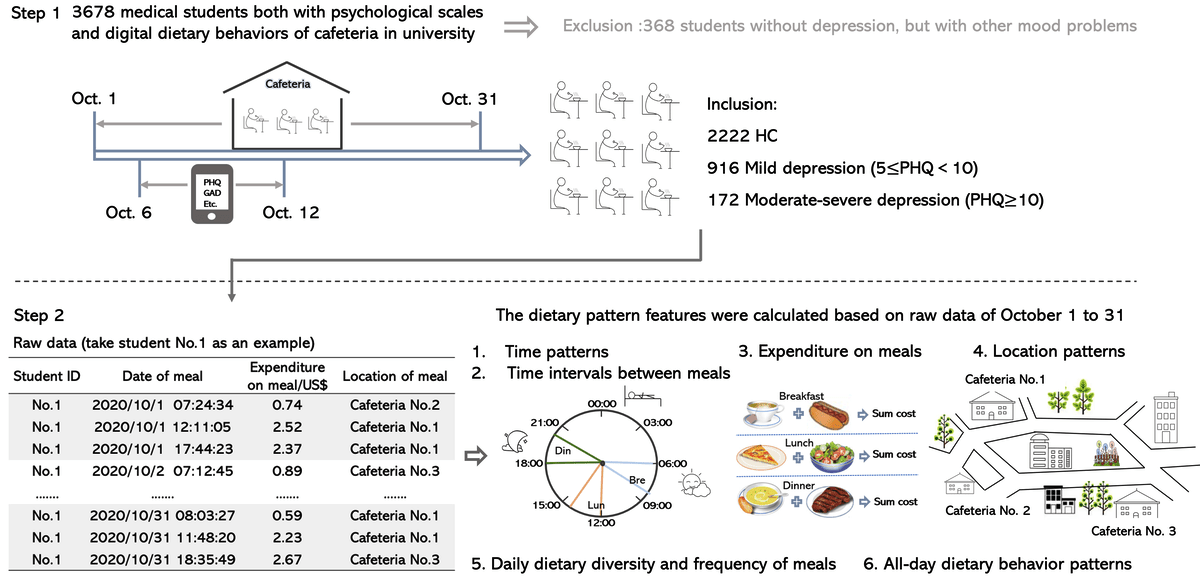
Flow diagram of the study design. Din: dinner; GAD-7: Generalized Anxiety Disorder Questionnaire-7; HC: healthy control; Lun: lunch; PHQ-9: Patient Health Questionnaire-9.

Inclusion criteria were established as follows: individuals in the mild depression group had PHQ-9 scores between 5 and 9, while those in the moderate-severe depression group had PHQ-9 scores of 10 or higher. The HC group had PHQ-9 scores below 5, GAD-7 scores below 5, PSS-14 scores below 29, and ISI scores below 8. Exclusion criteria for all participants included PHQ-9 scores below 5 and any of the following conditions: GAD-7 scores of 5 or higher, PSS-14 scores of 29 or higher, or ISI scores of 8 or higher. The participant inclusion process for this study is depicted in Figure 1 (Step 1).

### Ethical Considerations

Prior to their involvement in the study, all participants provided their informed consent through a formally endorsed consent form. The study was approved by the Biomedical Ethics Committee of Xinxiang Medical University (XYLL-2020235).

### Dietary Data Collection

During the COVID-19 pandemic in China, students were required to stay on campus, resulting in most students eating at the campus cafeterias on a daily basis. These meals are often paid for using electronic transactions linked to a student’s account, and there are 3 cafeterias at the school. The dietary data preprocess can be found in Multimedia Appendix 1. Previous studies have shown that diet time, or chronotype, is related to circadian rhythms and mood [5], and location and periodicity of meals are related to depression symptoms [24]. Therefore, we evaluated 6 dietary behavior features, including time, location, expenditure, daily dietary items and frequency of meals, and all-day dietary behavior patterns, to objectively assess dietary behaviors in relation to depression in this study (Figure 1, Step 2).

### Calculation of Dietary Features

#### Time Patterns

To determine the number of daily meals, we counted the number of records starting from the first recorded meal. Dietary behaviors within 2 hours of this initial record were part of a single meal, which could be further divided into up to 1, 2, or 3 meals per day: breakfast, lunch, and dinner. In addition, based on the cafeteria’s hours of operation, the 3 meals are divided into time slots as follows: breakfast from 6:30 AM to 8:30 AM, lunch from 11 AM to 1 PM, and dinner from 5:30 PM to 7:30 PM. If there were multiple electronic transactions within a single meal, we used the timestamp of the first transaction as the time of the meal for analysis.

#### Time Intervals Between Meals

We used the times of breakfast, lunch, and dinner calculated from the time patterns to determine the time intervals between each pair of meals: breakfast and lunch, lunch and dinner, and breakfast and dinner.

#### Expenditure on Meals

After dividing meals into breakfast, lunch, and dinner based on the time patterns, we calculated the total cost for each meal by summing up multiple electronic transactions for a single meal. Expenditure on a single meal may indicate an individual’s appetite as it reflects the amount of food purchased.

#### Location Patterns

To determine location patterns of dietary behavior, we calculated the frequency of visits to each cafeteria by counting total number of cafeterias visited in October and calculated the frequency of the student’s visits to the cafeteria for breakfast, lunch, and dinner using the following formula:


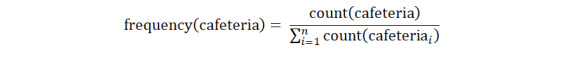


#### Daily Dietary Diversity and Frequency of Meals

The average number of different foods consumed per day in October was calculated as a measure of food diversity. The frequency of breakfast, lunch, and dinner in the month was also calculated as a measure of dietary habits. These measures were used to understand the dietary behaviors of participants in the study.

#### All-Day Dietary Behavior Patterns

People may have regular or irregular dietary behavior patterns, such as consistently or selectively eating breakfast, lunch, and dinner. There are 7 possible dietary behavior patterns: eating only breakfast, only lunch, only dinner, breakfast-lunch, lunch-dinner, and all 3 meals (breakfast-lunch-dinner pattern). All-day dietary behavior patterns are defined as the meals eaten by a participant on a given day. The frequency of a participant’s daily dietary behavior patterns can be calculated based on their dietary records for the entire month of October. To do this, the daily records are taken as a unit and the frequency of each dietary behavior pattern is calculated using the following formula:


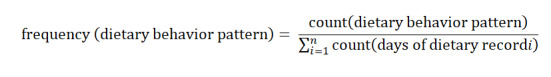


#### Statistical Indices of Dietary Features

Statistical indices were calculated for dietary features such as time patterns, intervals between meals, and expenditure on meals, including mean, median, median absolute deviation (MAD), and maximum and minimum values for the entire month of October. The study also separated weekday and weekend behavior by calculating dietary features separately for the 2 time periods. More information can be found in Table S1 in Multimedia Appendix 1.

### Statistical Analysis

ANOVA and chi-square tests were used to analyze demographic and psychological characteristics. ANCOVA (analyses of covariance) was used to compare dietary behavior features among groups, with age, gender, BMI, and educational level as covariates. All variables in the 6 dietary features were transformed into Z-scores to give equal weight and minimize the impact of outliers in the study. Logistic regression analyses were used to examine associations between all-day dietary behavior patterns and depression severity (mild depression, and moderate-severe depression), as well as associations between other psychological symptoms in depression groups or all participants and all-day dietary behavior patterns, with age, gender, BMI, and educational level as covariates. The all-day dietary behavior patterns were categorized into tertiles: Rare (less than 1 SD), Normal (within 1 SD), and Always (more than 1 SD) in logistic regression analyses. Mediation analysis was performed using Model 4 with 1 independent variable (all-day dietary behavior patterns), 1 dependent variable (groups), and 1 moderator (other psychological symptoms) in the PROCESS. A bootstrapping procedure with 95% CIs was used to measure the moderating effect, with 5000 bootstrap samples. Age, gender, BMI, and educational level were included as covariates in the model. All analyses were conducted using SPSS (version 25.0; IBM Corp).

### Classification Model

We analyzed changes in dietary behaviors between individuals with HC and depression to detect depression. These dietary behaviors were divided into tertile levels (HC, mild depression, and moderate-severe depression) and binary levels (HC and moderate-severe depression). We used a support vector machine with a radial basis function kernel as the classifier and selected the optimal parameters (0.1 for C and 0.001 for gamma) through grid search. These same parameters were used in all subsequent experiments. To avoid overfitting the model and ensure the accuracy of our results, we used 5-fold cross-validation and divided the training and validation sets in a 4:1 ratio.

## Results

### Demographic and Clinical Characteristics of Participators

A total of 3310 students, consisting of 2222 HC, 916 mild depression, and 172 moderate-severe depression, met the inclusion criteria and were ultimately included in the study. And 368 students exhibited psychological symptoms, notably anxiety, insomnia, or abnormal stress, while concurrently not manifesting depressive symptoms. As a result, they were excluded from the study cohort. In the students with mild depression, the following rates were observed: 37.7% (n=345) with anxiety, 17.8% (n=163) with perceived abnormal stress, and 23.8% (n=218) with insomnia. In moderate-severe depressive students, the corresponding percentages were: 75% (n=129) with anxiety, 70.3% (n=121) with perceived abnormal stress, and 53.5% (n=92) with insomnia. The demographic and clinical characteristics of the participants can be found in Table 1.

**Table 1:** Demographic and clinical characteristics of the study population.

			HC^a^		Mild depression		Moderate-severe depression		F test or chi-square test (*df*)			*P* value	
**Demographic characteristics**													
	Participants, n	2222		916		172		N/A^b^			N/A		
	Age (years), mean (SD)	19.46 (1.49)		19.48 (1.35)		19.64 (1.52)		1.209 (2)			.3		
**Sex, n (%)**										7.631 (2)			.02
	Male	864 (38.88)		315 (34.39)		74 (43.02)							
	Female	1358 (61.12)		601 (65.61)		98 (56.97)							
**BMI, n (%)**										4.783 (4)			.31
	＜18.5	281 (12.65)		142 (15.50)		22 (12.79)							
	18.5-24	1625 (73.13)		653 (71.29)		126 (73.26)							
	＞24	316 (14.22)		121 (13.21)		24 (13.95)							
**Educational level^c^, n (%)**										2.342 (4)			.67
	Undergraduate	2190 (98.56)		908 (99.13)		170 (98.84)							
	Postgraduate	16 (0.72)		3 (0.33)		1 (0.58)							
	Doctoral candidate	7 (0.32)		2 (0.22)		1 (0.58)							
**Clinical characteristics**													
	PHQ-9^d^ score, mean (SD)	1.42 (1.41)		6.58 (1.33)		12.94 (3.31)		7000.113 (2)			<.001		
	GAD-7^e^ score, mean (SD)	0.49 (0.92)		3.60 (2.74)		7.47 (4.26)		1712.361 (2)			<.001		
	GAD-7score ＞ 5 (Yes), n (%)	0		345 (37.66)		129 (75)		N/A			N/A		
	PSS-14^f^ score, mean (SD)	15.77 (6.79)		23.08 (6.50)		32.41 (6.63)		767.972 (2)			<.001		
	PSS score ＞ 28 (Yes), n (%)	0		163 (26.33)		121 (70.35)		N/A			N/A		
	ISI^g^ score, mean (SD)	1.78 (1.86)		5.42 (3.46)		8.93 (5.79)		992.727 (2)			<.001		
	ISI score ＞ 8 (Yes), n (%)	0		218 (23.80)		92 (53.49)		N/A			N/A		

^a^HC: healthy control.

^b^N/A: not applicable.

^c^Information that was missing for some participants.

^d^PHQ-9: Patient Health Questionnaire-9.

^e^GAD-7: Generalized Anxiety Disorder Questionnaire-7.

^f^PSS-14: Perceived Stress Scale-14.

^g^ISI: Insomnia Severity Index.

### Dietary Features Among HC, Mild Depression, and Moderate-Severe Depression

The ANCOVA analyses showed that there were significant differences between the groups in terms of time patterns, the intervals between meals, expenditure on meals, daily dietary diversity and frequency of meals, and all-day dietary behavior patterns (details in Table S2 in Multimedia Appendix 1). There was no significant difference in location patterns among the 3 groups.

**Figure 2:**
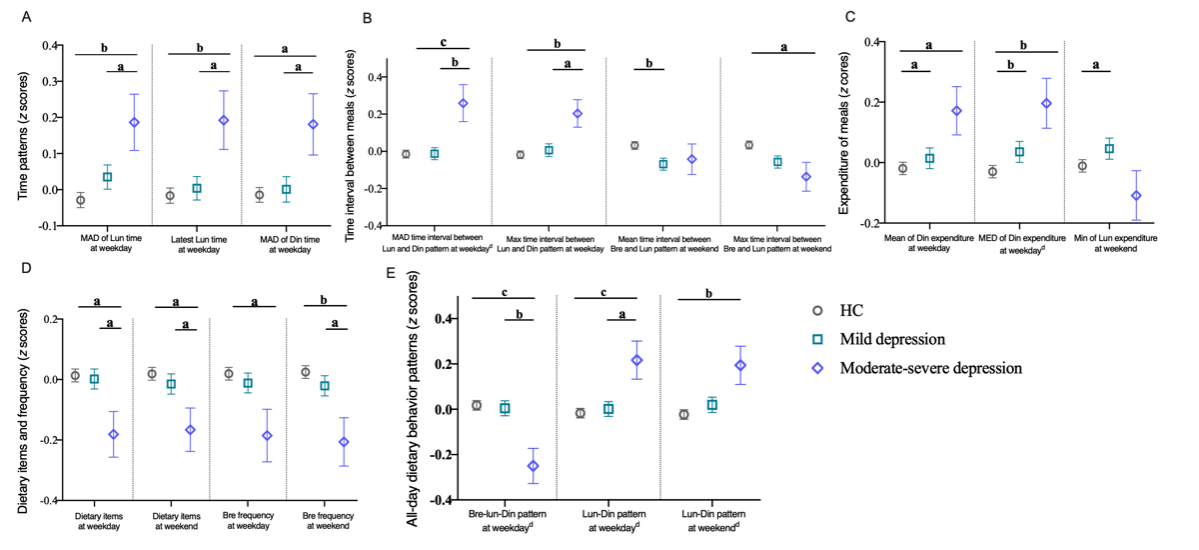
Alternations of dietary pattern features in mild depression and moderate-severe depression. a: *P*＜.05; b: *P*＜.01; c: *P*＜.001; and d: significance at *P*＜.05, after false discovery rate correction. The values of dietary pattern features in the graph were transformed into Z-scores. Bre: breakfast; Din: dinner; HC: healthy control; Lun: lunch; MAD: median absolute deviation; Max: maximum; MED: median; Min: minimum.

In terms of time patterns, post hoc analyses showed that compared with mild depression and HC, moderate-severe depression had significantly increased MAD of lunchtime (*P*=.04 and *P*=.004, separately), latest lunchtime (*P*=.02 and *P*=.007, separately) and MAD of dinner time (*P*=.02 and *P*=.01, separately) on weekdays, and there was no significant difference between mild depression and HC (Figure 2A). In terms of time intervals between meals, post hoc analyses that compared mild depression and HC, moderate-severe depression had increased MAD (*P*=.001 and *P*<.001, separately) and maximum (*P*=.02 and *P*=.005, separately) time intervals between lunch and dinner on weekdays, respectively, but mild depression and HC had no significant difference with each other (Figure 2B). On weekends, post hoc analyses that compared with HC, mild depression had a significant decrease in the mean time interval between breakfast and lunch (*P*=.01), while mild depression and moderate-severe depression had significant decreases in the maximum time interval between breakfast and lunch (*P*=.02 and *P*=.03 separately; Figure 2B). The MAD of lunchtime on weekdays was higher in moderate-severe depression (18 minutes 58 seconds) than mild depression (17 minutes 38 seconds) and HC (17 minutes 5 seconds). Similarly, the MAD of dinner time on a weekday was higher in moderate-severe depression (26 minutes 11 seconds) than in mild depression (23 minutes 39 seconds) and HC (23 minutes 26 seconds). Additionally, the MAD time interval between lunch and dinner on weekdays was larger in moderate-severe depression (34 minutes 20 seconds) than in mild depression (29 minutes 25 seconds) and HC (29 minutes 23 seconds; Table S2 in Multimedia Appendix 1). These findings indicate a temporally erratic lunch and dinner dietary pattern in moderate-severe depression.

In terms of expenditure on meals, compared with HC, both students with mild and moderate-severe depression had significant increases in the mean (*P*=.02 and *P*=.02, separately) and median (*P*=.002 and *P*=.006, separately) of dinner expenditure on weekdays (Figure 2C). However, there was no significant difference between mild depression and moderate-severe depression. On weekends, the mild depressive students had a significant increase in the minimum lunch expenditure compared with HC (*P*=.03) and moderate-severe depression (*P*=.01), but HC and moderate-severe depression had no significant difference from each other (Figure 2C).

In terms of daily dietary diversity, post hoc analyses that moderate-severe depression had significant decreases compared with mild depression and HC both on weekdays (*P*=.02 and *P*=.01, separately) and weekends (*P*=.03 and *P*=.01, separately). However, there was no significant difference between mild depression and HC. We also found that compared with HC, moderate-severe depression had a significant reduction in breakfast frequency on weekdays (*P*=.02) and weekends (*P*=.005). Additionally, compared with mild depression, moderate-severe depression had a significant reduction in breakfast frequency on weekends (*P*=.05). However, there was no significant difference between mild depression and HC in breakfast frequency on weekdays and weekends (Figure 2D).

Finally, in terms of all-day dietary behavior patterns, compared with HC and mild depression, moderate-severe depression had a reduction in the breakfast-lunch-dinner pattern on weekdays (*P*=.001 and *P*=.002, separately) and enrichment in the lunch-dinner pattern on weekdays (*P*=.005 and *P*=.03 separately; Figure 2E). However, there was no significant difference between HC and mild depression. On weekends, we observed that moderate-severe depression had an enrichment in the lunch-dinner pattern compared with HC (*P*=.008; Figure 2E).

### Associations Between All-Day Dietary Behavior Patterns and Depression

**Figure 3:**
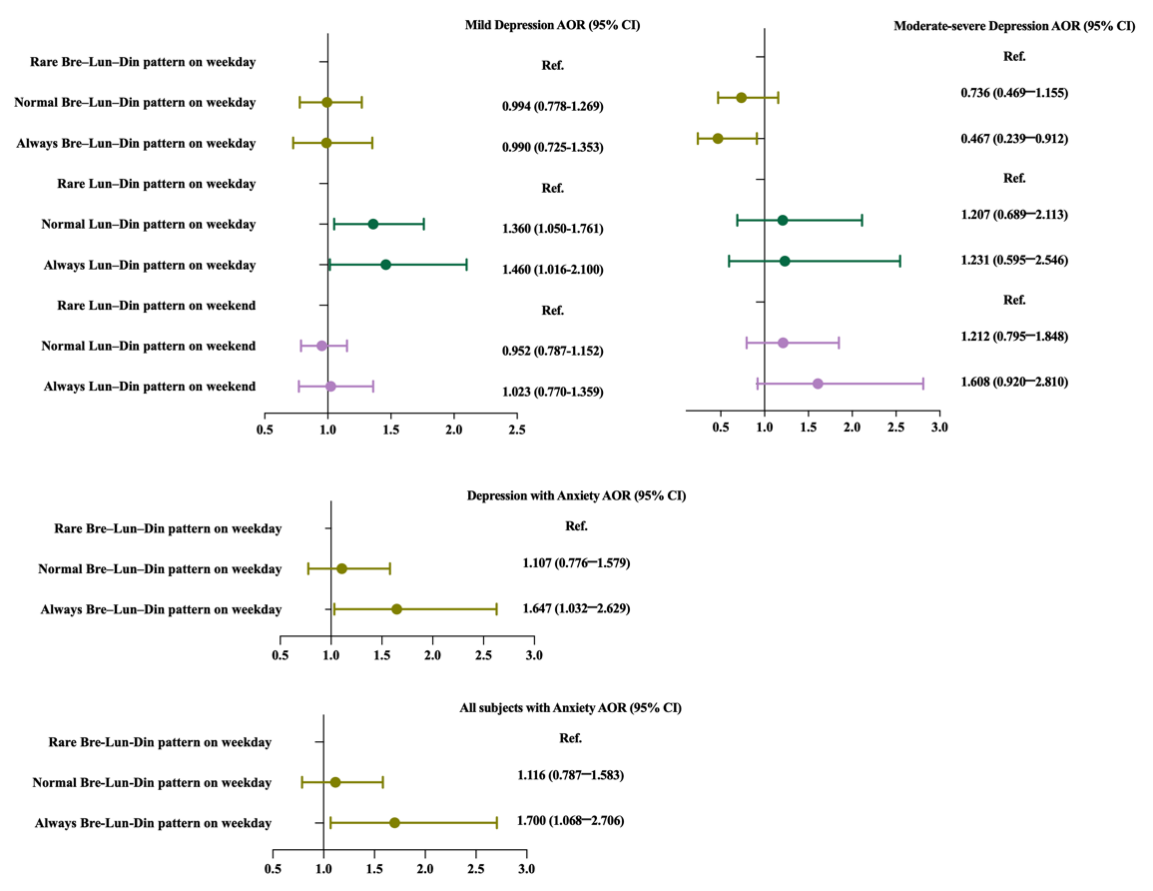
(A,B) Associations between all-day dietary behavior patterns and depression. (C) Associations between Bre-Lun-Din patterns on weekdays and comorbidity of depression and anxiety in individuals. (D) Associations between Bre-Lun-Din patterns on weekday and anxiety in individuals. (E) The mediating role of anxiety between groups (HC, mild depression, and moderate-severe depression) and Bre-Lun-Din patterns. a: *P*＜.05, b: *P*＜.01, and c: *P*＜.001. AOR: adjusted odds ratio; Bre: breakfast; Din: dinner; HC: healthy control; Lun: lunch.

To gain a deeper understanding of the relationship between comprehensive indicators of all-day dietary behavior patterns and depression severity. Using multiple logistic regression, we analyzed the associations between alterations in all-day dietary behavior patterns and depression severity in Figures 3A and 3B. After adjusting for age, gender, BMI, and educational level, we found that the Normal and Always lunch-dinner patterns on weekdays were positively associated with mild depression (Exp(B), 95% CI 1.360, 1.050-1.761; 1.460, 1.016-2.100; respectively), and the Always breakfast-lunch-dinner pattern on weekdays was negatively associated with moderate-severe depression (Exp(B), 95% CI 0.467, 0.239-0.912). The comparison of tertile levels of these all-day dietary behavior patterns among 3 groups can be found in Table S3 in Multimedia Appendix 1, and the percent of tertile levels of these all-day dietary behavior patterns can be found in Multimedia Appendix Figure S1.

### Associations Between All-Day Dietary Behavior Patterns and Other Clinical Symptoms

Our findings revealed a positive correlation between anxiety and adherence to the consistent always breakfast-lunch-dinner pattern on weekdays among individuals with depression as well as all participants (Figures 3C and 3D). However, no significant associations were discovered between other all-day dietary patterns and other clinical symptoms (Table S4 in Multimedia Appendix 1). In the mediation model, anxiety served as a mediator (*R*^2^=0.570, *P*<.001). A bootstrapped 95% CI confirmed that the indirect effect of groups (HC, mild depression, and moderate-severe depression) had an impact of 0.042 that was produced by anxiety as a mediator on breakfast-lunch-dinner pattern (Figure 3E).

### The Detection of Dietary Patterns for Depression

Our results showed that alterations in dietary behaviors of depression had an accuracy of 0.53, *F*_1_-score of 0.52, precision of 0.60, recall of 0.62, and an area under curve of 0.59 for detecting mild depression and moderate-severe depression. For detecting moderate-severe depression specifically, the accuracy was 0.67, *F*_1_-score was 0.60, precision was 0.64, recall was 0.65, and area under curve was 0.69 (Table 2).

**Table 2:** The detection of depression in classification models.

	Accuracy	*F*_1_-score	Precision	Recall	AUC^a^
HC^b^ versus mild depression versus moderate-severe depression	0.53	0.52	0.60	0.62	0.59
HC versus moderate-severe depression	0.67	0.60	0.64	0.65	0.69

^a^AUC: area under the curve.

^b^HC: healthy control.

## Discussion

### Principal Results

This study is the first to investigate digital dietary patterns of individuals with mild and moderate-severe depression using electronic device–based monitoring. The results indicate that individuals with moderate-severe depression exhibit more irregular eating time patterns, greater fluctuations in their feeding window, higher expenditure on dinner, lower food diversity, and a decreased frequency of consuming breakfast, as well as more irregular lunch-dinner patterns and less regular breakfast-lunch-dinner patterns compared with HC. The study also found that maintaining a regular breakfast-lunch-dinner pattern is negatively associated with moderate-severe depression, and maintaining an irregular lunch-dinner pattern is positively associated with mild depression. Additionally, the presence of anxiety is positively associated with the breakfast-lunch-dinner pattern, and the severity of anxiety has an indirect effect on the relationship between depression and the breakfast-lunch-dinner pattern. Importantly, the study suggests that digital dietary features can be used to detect depression, particularly moderate-severe depression, indicating that quantified digital behavior could be a promising approach to the detection of depression.

### Comparison With Prior Work in Dietary Behavior of Depression

We found that individuals with moderate-severe depression exhibited a reduction in dietary diversity, a decreased frequency of consuming breakfast, and irregular timing for lunch and dinner. The loss of interest in pleasurable activities, including eating, is a core symptom of depression, which may explain the decreased dietary diversity in individuals with moderate-severe depression. The findings of decreased breakfast frequency in moderate-severe depression align with previous research, which has identified a significant association between skipped or infrequent breakfast and an increased risk for depression [25,26]. The frequency of lunch and dinner did not significantly contribute to depression [26,27]. This discrepancy may be attributed to the fact that individuals with depression tend to have worse moods in the morning, which may negatively impact their appetite for breakfast. Considering these findings, it is important to consider the specific meal that is skipped when implementing dietary interventions to prevent depression. The study found that individuals with mild and moderate-severe depression exhibited significant fluctuations in the timing of lunch and dinner, as well as an irregular time interval between these meals. These findings align with previous research that has identified the importance of the feeding window on mental health [28,29], with irregular eating time patterns being associated with an increased risk of mental health distress [30]. Additionally, this study is the first to investigate the expenditure on 3 meals in individuals with depression, which provides insight into their food intake and appetite for each meal. The results indicate that individuals with mild and moderate-severe depression spent more money on dinner, which is consistent with the observation that individuals with major depressive disorder tend to have higher food intake during dinner compared with breakfast and lunch [31]. Notably, this study is the first to report that depression is associated with a preference for higher expenditure on dinner, while also being accompanied by a low frequency of breakfast.

This study is the first to objectively quantify all-day dietary behavior patterns by integrating time patterns, frequency of meals, and other parameters, and it revealed that moderate-severe depression is associated with an increase in lunch-dinner patterns and a decrease in breakfast-lunch-dinner patterns. Previous research has demonstrated that consuming breakfast, lunch, and dinner every day can reduce the prevalence of first-onset depression in a 5-year follow-up [32]. The lunch-dinner pattern is associated with eating later in the day and a meta-analysis has indicated that depressed patients are more inclined to spend on food later in the day, a phenomenon known as eveningness chronotype [33]. The relationship between eveningness chronotype and depressive symptoms aligns with pre-existing theories of chronobiology, which suggest that circadian dysfunction can have adverse effects on psychological well-being [34,35]. Eveningness chronotype is associated with a higher likelihood of regularly skipping or postponing breakfast [36] and it is well recognized that eating breakfast plays an important role in lowering blood cortisol levels and disturbances in glucose metabolism, which may affect serotonin levels [37].

Prior research in the dietary patterns field, whether dietary nutrition, dietary frequency, or dietary chronotype studies, predominantly relied on questionnaires [38,39]. The use of electronic behavioral monitoring for evaluating dietary behavioral patterns is still in its nascent technological phase. The self-monitoring is a prevalent method for assessing electronic dietary behaviors in the study of mental health [40,41]. This approach predominantly entails participants proactively documenting their daily dietary intake using an electronic device, often an app. However, it is important to acknowledge that excessive self-recording of behaviors could potentially lead to fatigue and monotony. While a frequency ranging from 2 to 3 times per week proved to be acceptable and reasonable [42], it once again harbors the inherent issue of recall bias that was previously encountered. Moreover, the field of wearable device-based monitoring for tracking dietary behavior has witnessed notable advancements [43,44]. However, its application in the field of psychiatry remains unexplored to date. The study marks the pioneering use of objective electronic device–based monitoring, unveiling anomalous eating patterns among individuals with depression.

### The Associations Between Dietary Behavioral Patterns and Depression

The study found that maintaining a lunch-dinner pattern was positively associated with mild depression. Furthermore, maintaining a breakfast-lunch-dinner pattern was negatively associated with moderate-severe depression. These findings align with previous research that has identified eating breakfast as a health-promoting behavior [45] and a positive association between skipping breakfast and depressive symptoms [46]. Additionally, it highlights the importance of meal substitution for regular eating patterns as a positive association with emotional disorders [47]. Overall, these findings suggest that maintaining a regular dietary pattern can be considered a dietary strategy for depression prevention.

Furthermore, the study found that anxiety is positively associated with the breakfast-lunch-dinner pattern. We can infer from the result that individuals with anxiety tend to choose to regularly eat 3 meals in their daily life and that depression with increasing anxiety may lead to an increase in the breakfast-lunch-dinner eating behavior. Literature has also indicated that individuals with anxiety tend to engage in more overeating situations [48] and have a positive association between anxiety symptoms and emotional and external eating [49]. However, the study also indicates that those with depression tend to intake more in dinner and then postpone or skip breakfast the next day. The study also found that anxiety could mediate the relationship between depression and breakfast-lunch-dinner pattern, which indicates that students with anxiety tend to maintain a regular breakfast-lunch-dinner pattern rather than postponing or skipping breakfast. This highlights the need for careful consideration when implementing dietary interventions for the comorbidity of depression and anxiety.

### The Potential Value of Digital Dietary Behaviors in Depression

This study is the first to use digital dietary behaviors based on real-world behavioral monitoring to detect mild depression and moderate-severe depression. The results indicate that digital dietary behaviors could distinguish between moderate-severe depression and HC. Depression is a disorder characterized by brain-based dysfunction that is expressed through behavioral changes. The diagnosis of depression traditionally relies on structured interviews or questionnaires, which are based on retrospective self-reports and the threshold scores of questionnaires. These methods can be prone to bias and subjectivity. The use of digital markers of continuous daily behavioral monitoring as an objective indicator to detect depression represents a promising supplementary approach [13].

In this study, students’ dietary behaviors were analyzed over a period of 1 month to draw a profile of dietary patterns in depression. This is the first exploration of the relationship between digital dietary behaviors and depression, and the use of digital dietary behaviors to detect depression. Another thing to watch out for is that the results indicate that a deep learning model of dietary behaviors from digital devices holds more accurate detection of moderate-severe depression than mild depression. This is likely due to the observation that moderate-severe depression presents more erratic dietary behaviors than mild depression and HCs. Overall, this study highlights the potential of digital dietary features as a promising manner in the detection of depression, particularly moderate-severe depression.

### Limitations

There are several limitations in this study that should be acknowledged. First, the measurement of depression severity in this study was based on self-reported data, future studies should consider clinician-based rating scales to reduce bias. Additionally, this study used a cross-sectional survey of depression, which does not capture changes in symptomology over time. A longitudinal psychological survey combined with continuous daily behavioral monitoring could provide further insight into dietary progression markers related to the severity of depressive symptoms over time. Finally, the study did not assess daily dietary structure and nutrient intake.

### Conclusions

This study represents a pioneering endeavor in objectively characterizing the digital dietary behaviors of individuals experiencing depression, using real-world monitoring as opposed to self-reported retrospective data. Our findings indicate that students with depression experience disruptions in various aspects, including time patterns, the intervals between meals, expenditure on meals, daily dietary diversity and frequency of meals, and all-day dietary behavior patterns. Notably, individuals with moderate-severe depression showcase greater irregularities in eating time patterns, fluctuated feeding windows, decreased food diversity, higher expenditure on dinner, and a preference for consuming only lunch and dinner. Furthermore, maintaining a regular breakfast-lunch-dinner eating pattern exhibits a negative correlation with moderate-severe depression. Keeping a lunch-dinner pattern is positively associated with mild depression. This research not only fills a critical gap in the existing academic literature but also sheds light on the promising potential of digital dietary behaviors as objective makers to the detection of depression, particularly moderate-severe depression.

## Appendix

Multimedia Appendix 1 Supplemental materials.

## Abbreviations

ANCOVA: analyses of covariance
GAD-7: Generalized Anxiety Disorder Questionnaire-7
HC: healthy control
ISI: Insomnia Severity Index
MAD: median absolute deviation
PHQ-9: Patient Health Questionnaire-9
PSS-14: Perceived Stress Scale-14

## Data Availability

The data sets generated and analyzed during this study are available from the co-corresponding authors (FW: fei.wang@yale.edu; XZZ: zhangxizhe@njmu.edu.cn) on reasonable request.
