# Supplementary material for: Digital Dietary Behaviors in Individuals With Depression: Real-World Behavioral Observation
Source: JMIR Public Health Surveill. 2024 Apr 22;10:e47428. doi: 10.2196/47428 (PMC11074900; doi:10.2196/47428)
Supplement: Multimedia Appendix 1 [file publichealth_v10i1e47428_app1.docx]

**Materials**

**M1: Psychological health measurements**

The Patient Health Questionnaire-9 (PHQ-9) is a self-report questionnaire that measures symptoms of depression. It includes 9 items scored on a 4-point scale ranging from 0 to 3. The total score ranges from 0 to 27, and scores below 5 are used to identify participants without depressive symptoms.

The Generalized Anxiety Disorder Questionnaire-7 (GAD-7) is used to evaluate the severity of anxiety symptoms. It includes 7 items scored on a 4-point scale ranging from 0 to 3. The total score ranges from 0 to 21, and scores below 5 are considered to indicate the absence of anxiety symptoms in participants.

The Perceived Stress Scale-14 (PSS-14) is a self-report questionnaire that assesses perceived stress. It includes 14 items scored on a 5-point scale ranging from 0 to 4. The total score ranges from 0 to 56, and scores below 29 are considered to indicate normal levels of perceived stress in participants.

The Insomnia Severity Index (ISI) is used to evaluate the severity of insomnia, including 7 items. Participants answer questions on a 5-point scale ranging from 0 to 4. The total score ranges from 0 to 28, and scores below 8 are defined as indicating the absence of insomnia in participants.

**M2: Dietary data preprocess**

We conducted a thorough data cleaning process to exclude participants with insufficient dietary data. The standards for acceptable data were as follows: participants had at least 16 days of dietary records; the interval between dietary records was no more than 3 days; and participants had a total of at least 47 dietary records throughout the month.

**Table S1 Dietary features**

| Dietary features | Calculate method |
| --- | --- |
| Time patterns | The mean, median, absolute median of dietary time, latest time and earliest time of eating were counted for breakfast, lunch and dinner. |
| Time intervals between meals | Mean, median, median absolute deviation, longest temporal interval, and shortest time interval between breakfast-lunch, lunch-dinner, and breakfast-dinner. |
| Expenditure on meals | The mean, median, absolute median difference in food spending, the most and least amount spent on food. |
| Location patterns | The frequency of the student's visit each cafeteria in breakfast, lunch, and dinner in a month. |
| Daily dietary items and frequency of meals | The numbers of food diversity of the day and the frequency of breakfast, lunch and dinner in a month. |
| All-day dietary behavior patterns | The frequency of only breakfast, only lunch, only dinner, Breakfast-Lunch, Lunch-Dinner, and Breakfast-Lunch-Dinner in a month. |

The dietary features were reported both at weekday (Monday to Friday) and weekend (Saturday or Sunday).

The mean and median value of the behavior sequence represents its average level and the data-concentrated trend, respectively. The median absolute deviation represents the data variation indicator. The extreme value of the behavior sequence is also important for the discovery of depressive-associated dietary features. For example, the earliest/latest eating time describe the unusual situations of dietary behavior, the maximum/minimum dietary cost describe the individual of best/worst appetite situation. The maximum/minimum frequency of the dietary location represent whether the participant prefers to eating at the same cafeteria or changing cafeteria often. Therefore, the maximum/minimum values of statistical indexes represent the most extreme cases of the participant’s dietary behavior.

**Table S2 Alternations of dietary pattern features in mild depression and moderate-severe depression**

|  | HC | | Mild depression | | Moderate-severe depression | | F value | *P* value |
| --- | --- | --- | --- | --- | --- | --- | --- | --- |
|  | Mean | SD | Mean | SD | Mean | SD |  |  |
| **Dietary features on Weekday** | | | | | | | | |
| **Time patterns/s or hh:mm:ss** | | | | | | | | |
| Mean of breakfast time | 27515.14 | 3813.90 | 27395.52 | 4287.07 | 27506.59 | 5045.21 | 0.353 | .702 |
|  | 07:38:35 | 01:03:34 | 07:36:36 | 01:11:27 | 07:38:27 | 01:24:05 |  |  |
| MAD of breakfast time | 867.48 | 733.73 | 822.82 | 709.05 | 880.14 | 777.75 | 0.878 | .416 |
|  | 00:14:27 | 00:12:14 | 00:13:43 | 00:11:49 | 00:14:40 | 00:12:58 |  |  |
| MED of breakfast time | 27035.82 | 3836.55 | 26915.41 | 4297.37 | 26984.30 | 5035.51 | 0.330 | .719 |
|  | 07:30:36 | 01:03:57 | 07:28:35 | 01:11:37 | 07:29:44 | 01:23:56 |  |  |
| Min of breakfast time | 25007.83 | 3522.04 | 24932.42 | 3917.67 | 25083.52 | 4685.15 | 0.300 | .741 |
|  | 06:56:48 | 00:58:42 | 06:55:32 | 01:05:18 | 06:58:04 | 01:18:05 |  |  |
| Max of breakfast time | 31548.50 | 4762.56 | 31437.55 | 5212.29 | 31703.23 | 6047.46 | 0.383 | .682 |
|  | 08:46 | 01:19 | 08:44 | 01:27 | 08:49 | 01:41 |  |  |
| Mean of lunch time | 42286.98 | 997.24 | 42279.86 | 1002.45 | 42291.92 | 1013.10 | 0.019 | .981 |
|  | 11:44:47 | 00:16:37 | 11:44:40 | 00:16:42 | 11:44:52 | 00:16:53 |  |  |
| MAD of lunch time | 1024.74 | 518.67 | 1058.27 | 533.76 | 1137.63 | 535.34 | 4.805 | .008 |
|  | 00:17:05 | 00:08:39 | 00:17:38 | 00:08:54 | 00:18:58 | 00:08:55 |  |  |
| MED of lunch time | 42298.16 | 1182.05 | 42274.14 | 1187.04 | 42228.52 | 1228.30 | 0.406 | .666 |
|  | 11:44:58 | 00:19:42 | 11:44:34 | 00:19:47 | 11:43:49 | 00:20:28 |  |  |
| Min of lunch time | 38901.27 | 1516.46 | 38894.37 | 1556.21 | 38676.45 | 1543.60 | 2.289 | .101 |
|  | 10:48:21 | 00:25:16 | 10:48:14 | 00:25:56 | 10:44:36 | 00:25:44 |  |  |
| Max of lunch time | 46289.15 | 2811.42 | 46346.87 | 2755.82 | 46876.06 | 2989.31 | 3.618 | .027 |
|  | 12:51:29 | 00:46:51 | 12:52:27 | 00:45:56 | 13:01:16 | 00:49:49 |  |  |
| Mean of dinner time | 65089.43 | 2511.26 | 65046.06 | 4076.92 | 65374.12 | 1656.94 | 0.606 | .546 |
|  | 18:04:49 | 00:41:51 | 18:04:06 | 01:07:57 | 18:09:34 | 00:27:37 |  |  |
| MAD of dinner time | 1406.13 | 812.80 | 1419.15 | 900.10 | 1571.27 | 940.78 | 3.363 | .035 |
|  | 00:23:26 | 00:13:33 | 00:23:39 | 00:15:00 | 00:26:11 | 00:15:41 |  |  |
| MED of dinner time | 64775.38 | 2480.72 | 64766.04 | 4044.94 | 65089.90 | 1697.32 | 0.754 | .471 |
|  | 17:59:35 | 00:41:21 | 17:59:26 | 01:07:25 | 18:04:50 | 00:28:17 |  |  |
| Min of dinner time | 61263.08 | 2480.70 | 61117.98 | 3960.77 | 61033.61 | 1871.61 | 0.923 | .398 |
|  | 17:01:03 | 00:41:21 | 16:58:38 | 01:06:01 | 16:57:14 | 00:31:12 |  |  |
| Max of dinner time | 70432.70 | 4406.79 | 70440.96 | 5603.27 | 70929.40 | 3890.52 | 0.786 | .456 |
|  | 19:33:53 | 01:13:27 | 19:34:01 | 01:33:23 | 19:42:09 | 01:04:51 |  |  |
| **Time intervals between meals/s or hh:mm:ss** | | | | | | | | |
| Mean time interval between breakfast and lunch | 16611.30 | 3681.65 | 16365.18 | 3690.91 | 16093.28 | 4139.23 | 2.532 | .080 |
|  | 04:36:51 | 01:01:22 | 04:32:45 | 01:01:31 | 04:28:13 | 01:08:59 |  |  |
| MAD time interval between breakfast and lunch | 1912.02 | 1457.72 | 1890.27 | 1407.23 | 1990.70 | 1561.88 | 0.291 | .747 |
|  | 00:31:52 | 00:24:18 | 00:31:30 | 00:23:27 | 00:33:11 | 00:26:02 |  |  |
| MED time interval between breakfast and lunch | 15101.68 | 3175.35 | 14928.22 | 3174.94 | 14714.83 | 3865.64 | 1.833 | .160 |
|  | 04:11:42 | 00:52:55 | 04:08:48 | 00:52:55 | 04:05:15 | 01:04:26 |  |  |
| Min time interval between breakfast and lunch | 10180.18 | 3022.12 | 10115.22 | 2939.72 | 10046.32 | 3281.27 | 0.296 | .744 |
|  | 02:49:40 | 00:50:22 | 02:48:35 | 00:49:00 | 02:47:26 | 00:54:41 |  |  |
| Max time interval between breakfast and lunch | 30624.67 | 10846.68 | 30229.66 | 11258.76 | 29600.59 | 11473.57 | 0.942 | .390 |
|  | 08:30:25 | 03:00:47 | 08:23:50 | 03:07:39 | 08:13:21 | 03:11:14 |  |  |
| Mean time interval between lunch and dinner | 22504.80 | 1990.52 | 22599.96 | 2312.90 | 22804.68 | 1832.27 | 2.214 | .109 |
|  | 06:15:05 | 00:33:11 | 06:16:40 | 00:38:33 | 06:20:05 | 00:30:32 |  |  |
| MAD time interval between lunch and dinner | 1763.12 | 1062.08 | 1765.48 | 1062.52 | 2060.26 | 1416.19 | 6.483 | .002 |
|  | 00:29:23 | 00:17:42 | 00:29:25 | 00:17:43 | 00:34:20 | 00:23:36 |  |  |
| MED time interval between lunch and dinner | 22494.32 | 1990.91 | 22579.32 | 2328.24 | 22825.88 | 1885.97 | 2.397 | .091 |
|  | 06:14:54 | 00:33:11 | 06:16:19 | 00:38:48 | 06:20:26 | 00:31:26 |  |  |
| Min time interval between lunch and dinner | 16971.39 | 4494.97 | 17052.62 | 4569.62 | 16794.81 | 4641.24 | 0.306 | .736 |
|  | 04:42:51 | 01:14:55 | 04:44:13 | 01:16:10 | 04:39:55 | 01:17:21 |  |  |
| Max time interval between lunch and dinner | 27774.51 | 3711.07 | 27867.16 | 3954.21 | 28612.90 | 3664.65 | 3.981 | .019 |
|  | 07:42:55 | 01:01:51 | 07:44:27 | 01:05:54 | 07:56:53 | 01:01:05 |  |  |
| **Expenditure on meals/Yuan (CNY)** | | | | | | | | |
| Mean of breakfast expenditure | 4.02 | 1.48 | 3.99 | 1.57 | 4.01 | 2.05 | 0.269 | .764 |
| MAD of breakfast expenditure | 0.68 | 0.63 | 0.67 | 0.58 | 0.66 | 0.57 | 0.174 | .840 |
| MED of breakfast expenditure | 3.80 | 1.52 | 3.76 | 1.55 | 3.74 | 2.18 | 0.443 | .642 |
| Min of breakfast expenditure | 2.12 | 1.04 | 2.11 | 1.24 | 2.18 | 1.50 | 0.338 | .713 |
| Max of breakfast expenditure | 7.31 | 3.88 | 7.38 | 4.08 | 7.30 | 4.15 | 0.531 | .588 |
| Mean of lunch expenditure | 9.10 | 1.90 | 9.13 | 2.00 | 9.02 | 1.94 | 1.536 | .215 |
| MAD of lunch expenditure | 1.29 | 0.91 | 1.29 | 0.89 | 1.30 | 0.91 | 0.074 | .929 |
| MED of lunch expenditure | 8.35 | 1.68 | 8.43 | 1.88 | 8.38 | 1.98 | 2.193 | .112 |
| Min of lunch expenditure | 4.49 | 2.08 | 4.53 | 2.09 | 4.45 | 2.08 | 0.708 | .493 |
| Max of lunch expenditure | 17.64 | 6.67 | 17.47 | 6.62 | 17.07 | 5.88 | 0.774 | .461 |
| Mean of dinner expenditure | 6.45 | 2.43 | 6.53 | 2.59 | 6.93 | 2.60 | 4.460 | .012 |
| MAD of dinner expenditure | 1.51 | 0.97 | 1.57 | 1.01 | 1.59 | 1.02 | 2.074 | .126 |
| MED of dinner expenditure | 5.86 | 2.32 | 6.02 | 2.51 | 6.40 | 2.59 | 7.285 | .001 |
| Min of dinner expenditure | 2.62 | 1.48 | 2.63 | 1.47 | 2.75 | 1.52 | 0.942 | .390 |
| Max of dinner expenditure | 13.52 | 7.28 | 13.54 | 6.57 | 14.28 | 6.67 | 0.950 | .387 |
| **Location patterns** | | | | | | | | |
| Frequency of visiting cafeteria in breakfast | 0.43 | 0.20 | 0.42 | 0.19 | 0.45 | 0.23 | 2.252 | .105 |
| Frequency of visiting cafeteria in lunch | 0.52 | 0.16 | 0.52 | 0.16 | 0.52 | 0.17 | 0.390 | .677 |
| Frequency of visiting cafeteria in dinner | 0.58 | 0.17 | 0.58 | 0.18 | 0.58 | 0.17 | 0.015 | .985 |
| **Daily dietary diversity and frequency of meals** | | | | | | | | |
| All-day dietary diversity | 46.22 | 7.63 | 46.13 | 7.61 | 44.74 | 7.54 | 3.338 | .036 |
| Frequency of breakfast | 0.30 | 0.10 | 0.30 | 0.10 | 0.28 | 0.12 | 3.522 | .030 |
| Frequency of lunch | 0.39 | 0.06 | 0.39 | 0.06 | 0.40 | 0.07 | 2.690 | .068 |
| Frequency of dinner | 0.31 | 0.09 | 0.31 | 0.09 | 0.32 | 0.10 | 1.270 | .281 |
| **All-day dietary behavior patterns** | | | | | | | | |
| Only breakfast pattern | 0.02 | 0.03 | 0.02 | 0.03 | 0.02 | 0.03 | 0.038 | .962 |
| Only lunch pattern | 0.05 | 0.05 | 0.05 | 0.05 | 0.05 | 0.05 | 1.396 | .248 |
| Only dinner pattern | 0.02 | 0.03 | 0.02 | 0.03 | 0.03 | 0.04 | 3.826 | .022 |
| Breakfast-lunch pattern | 0.11 | 0.08 | 0.11 | 0.08 | 0.11 | 0.09 | 1.150 | .317 |
| Lunch-dinner pattern | 0.09 | 0.10 | 0.10 | 0.10 | 0.12 | 0.11 | 4.184 | .015 |
| Breakfast-lunch-dinner pattern | 0.16 | 0.07 | 0.16 | 0.07 | 0.14 | 0.07 | 5.814 | .003 |
| **Dietary features on Weekend** | | | | | | | | |
| **Time patterns/s or hh:mm:ss** | | | | | | | | |
| Mean of breakfast time | 27598.29 | 6783.01 | 27358.79 | 7322.32 | 26523.84 | 8989.07 | 2.075 | .126 |
|  | 07:39:58 | 01:53:03 | 07:35:59 | 02:02:02 | 07:22:04 | 02:29:49 |  |  |
| MAD of breakfast time | 1052.80 | 890.36 | 1077.56 | 949.24 | 1046.06 | 1027.71 | 0.213 | .808 |
|  | 00:17:33 | 00:14:50 | 00:17:58 | 00:15:49 | 00:17:26 | 00:17:08 |  |  |
| MED of breakfast time | 27630.30 | 6855.26 | 27369.39 | 7388.82 | 26438.19 | 9019.63 | 2.501 | .082 |
|  | 07:40:30 | 01:54:15 | 07:36:09 | 02:03:09 | 07:20:38 | 02:30:20 |  |  |
| Min of breakfast time | 25391.14 | 6322.70 | 25135.50 | 6814.67 | 24440.77 | 8345.43 | 1.979 | .138 |
|  | 07:03:11 | 01:45:23 | 06:58:55 | 01:53:35 | 06:47:21 | 02:19:05 |  |  |
| Max of breakfast time | 29762.58 | 7440.97 | 29616.33 | 8049.47 | 28813.23 | 9866.44 | 1.253 | .286 |
|  | 08:16:03 | 02:04:01 | 08:13:36 | 02:14:09 | 08:00:13 | 02:44:26 |  |  |
| Mean of lunch time | 42273.65 | 1904.85 | 42347.31 | 1320.03 | 42397.02 | 1387.69 | 1.080 | .340 |
|  | 11:44:34 | 00:31:45 | 11:45:47 | 00:22:00 | 11:46:37 | 00:23:08 |  |  |
| MAD of lunch time | 1068.67 | 705.00 | 1114.92 | 731.29 | 1147.69 | 718.05 | 1.893 | .151 |
|  | 00:17:49 | 00:11:45 | 00:18:35 | 00:12:11 | 00:19:08 | 00:11:58 |  |  |
| MED of lunch time | 42153.33 | 1963.17 | 42257.59 | 1426.85 | 42354.26 | 1547.43 | 2.153 | .116 |
|  | 11:42:33 | 00:32:43 | 11:44:18 | 00:23:47 | 11:45:54 | 00:25:47 |  |  |
| Min of lunch time | 39706.18 | 2029.98 | 39762.17 | 1663.06 | 39626.48 | 1698.61 | 0.874 | .418 |
|  | 11:01:46 | 00:33:50 | 11:02:42 | 00:27:43 | 11:00:26 | 00:28:19 |  |  |
| Max of lunch time | 45187.18 | 3223.24 | 45279.12 | 2839.24 | 45333.89 | 2629.23 | 0.401 | .669 |
|  | 12:33:07 | 00:53:43 | 12:34:39 | 00:47:19 | 12:35:34 | 00:43:49 |  |  |
| Mean of dinner time | 64807.59 | 7458.88 | 64663.58 | 8008.92 | 65043.07 | 7327.07 | 0.104 | .901 |
|  | 18:00:08 | 02:04:19 | 17:57:44 | 02:13:29 | 18:04:03 | 02:02:07 |  |  |
| MAD of dinner time | 1298.49 | 1088.87 | 1283.94 | 1007.17 | 1265.51 | 985.71 | 0.166 | .847 |
|  | 00:21:38 | 00:18:09 | 00:21:24 | 00:16:47 | 00:21:06 | 00:16:26 |  |  |
| MED of dinner time | 64594.65 | 7458.16 | 64459.29 | 8004.94 | 64852.62 | 7311.69 | 0.114 | .893 |
|  | 17:56:35 | 02:04:18 | 17:54:19 | 02:13:25 | 18:00:53 | 02:01:52 |  |  |
| Min of dinner time | 62039.61 | 7175.75 | 61956.75 | 7756.89 | 62287.99 | 7136.64 | 0.076 | .926 |
|  | 17:14:00 | 01:59:36 | 17:12:37 | 02:09:17 | 17:18:08 | 01:58:57 |  |  |
| Max of dinner time | 68150.62 | 8450.98 | 67919.15 | 8878.56 | 68439.19 | 8331.11 | 0.197 | .821 |
|  | 18:55:51 | 02:20:51 | 18:51:59 | 02:27:59 | 19:00:39 | 02:18:51 |  |  |
| **Time intervals between meals/s or hh:mm:ss** | | | | | | | | |
| Mean time interval between breakfast and lunch | 15801.56 | 6493.11 | 15148.23 | 6359.58 | 15322.27 | 6998.94 | 3.588 | .028 |
|  | 04:23:22 | 01:48:13 | 04:12:28 | 01:46:00 | 04:15:22 | 01:56:39 |  |  |
| MAD time interval between breakfast and lunch | 2185.25 | 2753.80 | 2131.53 | 2634.37 | 2312.80 | 3240.15 | 0.316 | .729 |
|  | 00:36:25 | 00:45:54 | 00:35:32 | 00:43:54 | 00:38:33 | 00:54:00 |  |  |
| MED time interval between breakfast and lunch | 14611.75 | 6431.75 | 14078.85 | 6239.99 | 14501.23 | 6870.06 | 2.362 | .094 |
|  | 04:03:32 | 01:47:12 | 03:54:39 | 01:44:00 | 04:01:41 | 01:54:30 |  |  |
| Min time interval between breakfast and lunch | 11086.28 | 5234.11 | 10743.06 | 4903.15 | 10971.33 | 5653.20 | 1.606 | .201 |
|  | 03:04:46 | 01:27:14 | 02:59:03 | 01:21:43 | 03:02:51 | 01:34:13 |  |  |
| Max time interval between breakfast and lunch | 23186.64 | 12058.64 | 22087.36 | 11901.93 | 21139.91 | 12251.20 | 4.469 | .012 |
|  | 06:26:27 | 03:20:59 | 06:08:07 | 03:18:22 | 05:52:20 | 03:24:11 |  |  |
| Mean time interval between lunch and dinner | 22010.34 | 4861.60 | 22112.18 | 4581.42 | 22301.05 | 4434.90 | 0.476 | .621 |
|  | 06:06:50 | 01:21:02 | 06:08:32 | 01:16:21 | 06:11:41 | 01:13:55 |  |  |
| MAD time interval between lunch and dinner | 1556.19 | 1639.94 | 1498.63 | 1606.25 | 1504.34 | 1468.99 | 0.483 | .617 |
|  | 00:25:56 | 00:27:20 | 00:24:59 | 00:26:46 | 00:25:04 | 00:24:29 |  |  |
| MED time interval between lunch and dinner | 22171.18 | 4815.28 | 22280.98 | 4534.04 | 22538.23 | 4515.53 | 0.672 | .511 |
|  | 06:09:31 | 01:20:15 | 06:11:21 | 01:15:34 | 06:15:38 | 01:15:16 |  |  |
| Min time interval between lunch and dinner | 18363.04 | 6327.64 | 18669.80 | 5859.42 | 18644.35 | 6195.58 | 0.910 | .403 |
|  | 05:06:03 | 01:45:28 | 05:11:10 | 01:37:39 | 05:10:44 | 01:43:16 |  |  |
| Max time interval between lunch and dinner | 25248.88 | 5859.56 | 25197.51 | 5537.50 | 25412.69 | 5182.16 | 0.043 | .958 |
|  | 07:00:49 | 01:37:40 | 06:59:58 | 01:32:17 | 07:03:33 | 01:26:22 |  |  |
| **Expenditure on meals** | | | | | | | | |
| Mean of breakfast expenditure | 4.09 | 1.90 | 4.05 | 1.87 | 3.96 | 2.22 | 0.712 | .491 |
| MAD of breakfast expenditure | 0.61 | 0.82 | 0.63 | 0.76 | 0.57 | 0.70 | 0.724 | .485 |
| MED of breakfast expenditure | 3.90 | 1.83 | 3.88 | 1.78 | 3.74 | 2.28 | 1.056 | .348 |
| Min of breakfast expenditure | 2.76 | 1.54 | 2.78 | 1.58 | 2.65 | 1.45 | 1.341 | .262 |
| Max of breakfast expenditure | 5.92 | 3.53 | 5.79 | 3.30 | 5.69 | 3.92 | 0.591 | .554 |
| Mean of lunch expenditure | 9.48 | 2.42 | 9.61 | 2.52 | 9.45 | 2.54 | 2.331 | .097 |
| MAD of lunch expenditure | 1.35 | 1.25 | 1.34 | 1.22 | 1.36 | 1.20 | 0.027 | .974 |
| MED of lunch expenditure | 8.79 | 2.26 | 8.87 | 2.36 | 8.83 | 2.45 | 1.224 | .294 |
| Min of lunch expenditure | 6.20 | 1.85 | 6.31 | 2.02 | 6.02 | 2.05 | 4.151 | .016 |
| Max of lunch expenditure | 14.85 | 6.17 | 15.01 | 6.17 | 14.63 | 6.20 | 0.742 | .476 |
| Mean of dinner expenditure | 6.50 | 3.01 | 6.63 | 3.06 | 6.70 | 2.74 | 2.609 | .074 |
| MAD of dinner expenditure | 1.34 | 1.25 | 1.38 | 1.23 | 1.43 | 1.20 | 1.093 | .335 |
| MED of dinner expenditure | 6.08 | 2.94 | 6.19 | 2.95 | 6.25 | 2.61 | 2.005 | .135 |
| Min of dinner expenditure | 3.66 | 2.33 | 3.73 | 2.26 | 3.68 | 1.94 | 1.426 | .241 |
| Max of dinner expenditure | 10.55 | 5.85 | 10.69 | 5.75 | 10.74 | 5.33 | 1.179 | .308 |
| **Location patterns** | | | | | | | | |
| Frequency of visiting cafeteria in breakfast | 0.67 | 0.28 | 0.68 | 0.29 | 0.67 | 0.32 | 0.006 | .994 |
| Frequency of visiting cafeteria in lunch | 0.74 | 0.20 | 0.74 | 0.20 | 0.71 | 0.21 | 1.241 | .289 |
| Frequency of visiting cafeteria in dinner | 0.78 | 0.21 | 0.78 | 0.22 | 0.79 | 0.22 | 0.410 | .664 |
| **Daily dietary diversity and frequency of meals** | | | | | | | | |
| All-day dietary diversity | 16.61 | 4.15 | 16.47 | 4.21 | 15.84 | 3.91 | 3.233 | .040 |
| Frequency of breakfast | 0.27 | 0.13 | 0.27 | 0.14 | 0.24 | 0.14 | 4.647 | .010 |
| Frequency of lunch | 0.41 | 0.10 | 0.41 | 0.10 | 0.42 | 0.10 | 2.751 | .064 |
| Frequency of dinner | 0.32 | 0.11 | 0.32 | 0.11 | 0.34 | 0.12 | 1.065 | .345 |
| **All-day dietary behavior patterns** | | | | | | | | |
| Only breakfast pattern | 0.04 | 0.07 | 0.04 | 0.06 | 0.03 | 0.06 | 1.345 | .261 |
| Only lunch pattern | 0.08 | 0.10 | 0.08 | 0.10 | 0.10 | 0.10 | 1.900 | .150 |
| Only dinner pattern | 0.04 | 0.07 | 0.04 | 0.07 | 0.05 | 0.07 | 0.440 | .644 |
| Breakfast-lunch pattern | 0.11 | 0.10 | 0.11 | 0.10 | 0.10 | 0.10 | 1.808 | .164 |
| Lunch-dinner pattern | 0.12 | 0.11 | 0.12 | 0.12 | 0.14 | 0.13 | 4.139 | .016 |
| Breakfast-lunch-dinner pattern | 0.12 | 0.08 | 0.12 | 0.09 | 0.11 | 0.08 | 1.689 | .185 |

SD: Standard Deviation; MAD: median absolute deviation; MED: median; Min: minimum; Max: maximum; ^b^ indicates significance at *P* < .05, after FDR correction

**Table S3 The comparison of tertile-levels of all-day dietary behavior patterns among three groups**

|  | HC  N=2222 | Mild depression  N=916 | Moderate-severe depression  N=172 | х^2^test | *P* value |
| --- | --- | --- | --- | --- | --- |
| **Bre-Lun-Din pattern on weekday** | | | |  |  |
| Rare | 360 | 153 | 41 |  |  |
| Normal | 1516 | 627 | 115 |  |  |
| Always | 346 | 136 | 16 | 9.819 | .044 |
| **Lun-Din pattern on weekday** | | | | | |
| Rare | 296 | 95 | 16 |  |  |
| Normal | 1594 | 674 | 117 |  |  |
| Always | 332 | 147 | 39 | 12.857 | .012 |
| **Lun-Din pattern on weekend** | | | | | |
| Rare | 591 | 240 | 34 |  |  |
| Normal | 1301 | 527 | 98 |  |  |
| Always | 330 | 149 | 40 | 10.483 | .033 |

Data are presented as n. Bre: breakfast; Lun: lunch; Din: dinner.

**Table S4 Associations between clinical symptoms and traditional dietary pattern**

| **Clinical symptoms** | **Bre-Lun-Din pattern in weekday in all subjects^a^** | | | | |
| --- | --- | --- | --- | --- | --- |
|  | Rare | Normal | | Always | |
|  |  | Exp(B) | 95% CIs | Exp(B) | 95% CIs |
| Anxiety (Yes) | Ref. | 1.116 | 0.787-1.583 | 1.700 | 1.068-2.706 |
| Stress (Yes) | Ref. | 0.896 | 0.605-1.329 | 0.848 | 0.496-1.453 |
| Insomnia (Yes) | Ref. | 0.903 | 0.630-1.296 | 0.784 | 0.477-1.288 |
|  | **Bre-Lun-Din pattern in weekday in depression^a^** | | | | |
| Anxiety (Yes) | Ref. | 1.107 | 0.776-1.579 | 1.647 | 1.032-2.629 |
| Stress (Yes) | Ref. | 0.939 | 0.620-1.422 | 0.977 | 0.558-1.710 |
| Insomnia (Yes) | Ref. | 0.917 | 0.636-1.321 | 0.770 | 0.467-1.269 |

CIs: confidence intervals. a: Models are adjusted for age, gender, BMI, educational level. Bre: breakfast; Lun: lunch; Din: dinner


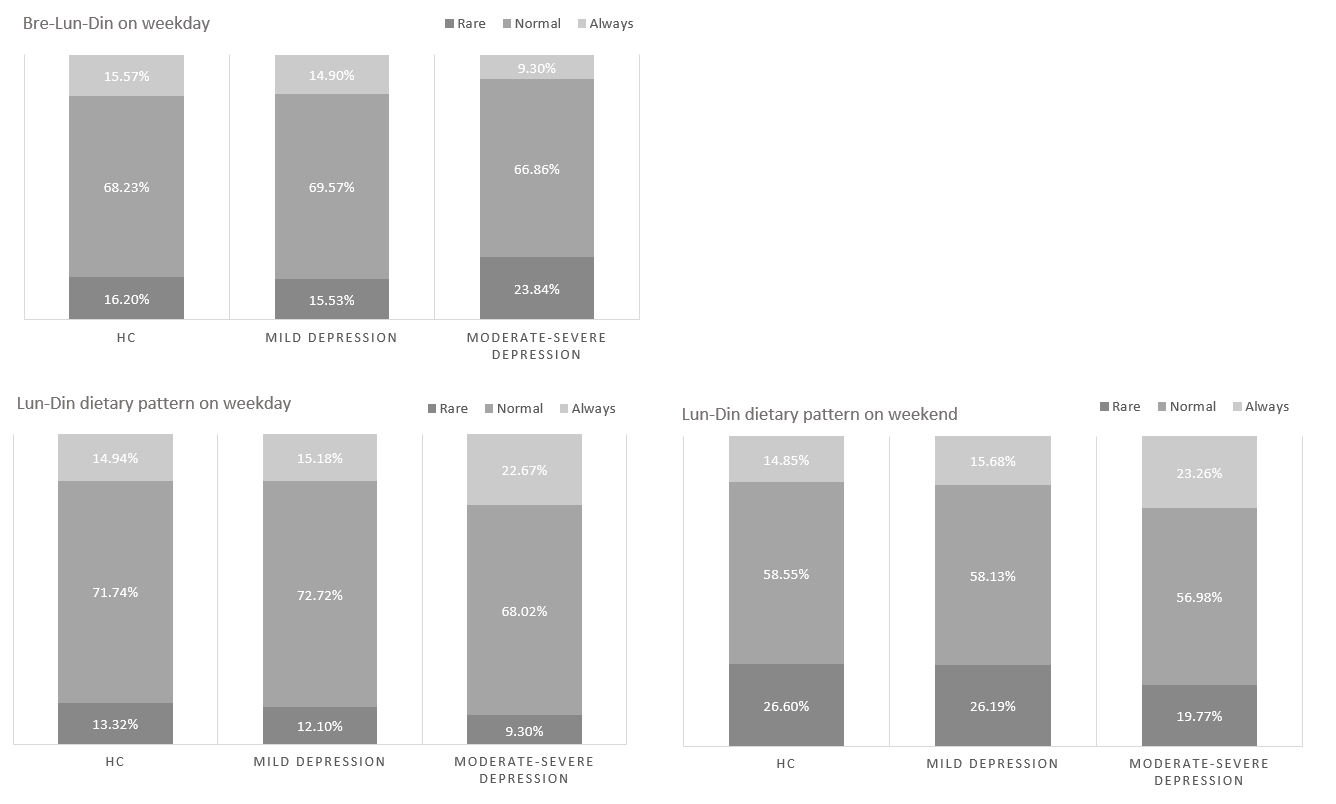


**Figure S1 The percent of tertile-levels of all-day dietary behavior patterns**
